# Physicochemical Properties, Antioxidant Capacity, and Sensory of Ready‐to‐Eat Flakes of Blue Corn and Purple Sweet Potato Blends

**DOI:** 10.1155/ijfo/3648714

**Published:** 2026-06-08

**Authors:** Betsabé Hernández-Santos, Jesús Rodríguez-Miranda, José M. Juárez-Barrientos, Juan G. Torruco-Uco, José M. Rivadeneyra-Rodríguez, Enrique Ramírez-Figueroa, Alma A. Lerdo-Reyes

**Affiliations:** ^1^ National Technological of Mexico/Technological Institute of Tuxtepec, Oaxaca, Mexico; ^2^ Institute of Agricultural Engineering, University of Papaloapan, Loma Bonita Campus, Oaxaca, Mexico, unpa.edu.mx

**Keywords:** antioxidant capacity, flakes, *Ipomoea batatas*, ready-to-eat, *Zea mays*

## Abstract

The physicochemical properties, antioxidant capacity, and sensory characteristics of ready‐to‐eat flakes formulated from blends of blue corn flour (BCF) and purple sweet potato flour (PSPF) were evaluated. Blending these raw materials represents a promising strategy for the development of functional ready‐to‐eat cereals due to their complementary composition, as blue corn provides phenolic compounds and carotenoids, whereas purple sweet potato contributes dietary fiber and anthocyanins with recognized antioxidant potential. Flakes were produced using 100% BCF, 100% PSPF, and blended formulations at ratios of 75/25, 50/50, and 25/75 (BCF/PSPF), respectively. Increasing the proportion of PSPF in the blends significantly enhanced dietary fiber content (3.03–8.72 g/100 g), water absorption capacity (2.49–2.89 g/g), milk solubility capacity (17.40%–30.04%), anthocyanin content (86.13–120.37 mg/100 g), and flavonoid content (35.35–42.95 mg catechin equivalents/g). Moreover, antioxidant activity increased as determined by DPPH• scavenging capacity (99.56% inhibition; IC_50_ = 1.83 mg/mL) and ABTS•^+^ scavenging activity (99.36% inhibition; IC_50_ = 0.63 mg/mL). Flakes formulated with 100% PSPF exhibited the highest true density (1.52 g/cm^3^) and breaking force (3.60 N). Conversely, increasing the proportion of BCF promoted higher protein content (3.25–5.63 g/100 g), water solubility index (4.37–30.87%), total polyphenol content (92.95–99.62 mg gallic acid equivalents/g), and *β*‐carotene content (4.08–7.55 mg *β*‐carotene/g). All flake formulations exhibited lower water activity than the commercial cereal and demonstrated reduced milk absorption capacity. Notably, flavonoids and total polyphenols were the most retained bioactive compounds after processing. Flakes containing higher levels of PSPF exhibited superior antioxidant capacity and achieved the highest sensory acceptability scores. Overall, ready‐to‐eat flakes formulated from whole BCF and PSPF blends exhibited significant antioxidant activity and physicochemical characteristics comparable with commercially available breakfast cereals. These results support their potential use as functional ingredients for the development of nutritionally enhanced ready‐to‐eat cereal products with added health‐promoting properties.

## 1. Introduction

In recent decades, the increasing prevalence of chronic noncommunicable diseases associated with oxidative stress has intensified interest in the development of functional foods enriched with bioactive compounds, particularly those exhibiting antioxidant capacity [1]. In this context, ready‐to‐eat breakfast cereals represent a strategic product category due to their widespread consumption and technological versatility. However, a considerable proportion of commercial products are primarily formulated with refined starch‐rich flours, thereby limiting their nutraceutical density. The incorporation of pigmented cereals, such as blue corn (*Zea mays* L.), has been proposed as an alternative strategy to enhance the phenolic compound content in cereal‐based matrices. Blue corn contains anthocyanins, phenolic acids, and other flavonoids that have been associated with higher antioxidant activity compared with conventional varieties [2, 3]. In addition, its high starch content promotes the formation of expandable matrices during thermal processing, a desirable characteristic for the production of snacks and flakes. Nevertheless, predominantly starchy formulations tend to generate highly porous structures with rapid liquid absorption, which may compromise textural stability during consumption with milk [4].

Purple sweet potato (*Ipomoea batatas* L.), in turn, is distinguished by its high content of acylated anthocyanins, flavonoids, and dietary fiber, compounds associated with strong antioxidant activity and relatively high thermal stability [5]. Unlike cereals, sweet potato presents a matrix rich in nonstarch polysaccharides and structural components capable of significantly modifying the physical and functional properties of the final product. In this regard, the incorporation of sweet potato flour into flake‐ or snack‐type products has been shown to increase antioxidant content while modulating attributes such as density, texture, and water absorption capacity (WAC) [6]. Additionally, previous studies have reported that purple sweet potato flour (PSPF) improves functional properties and enhances the bioactive compound content of cereal‐based products, contributing to increased antioxidant potential and nutritional quality, which supports its suitability as a functional ingredient in ready‐to‐eat formulations [7]. From a technological standpoint, the development of ready‐to‐eat flakes involves complex interactions among chemical composition, the degree of starch gelatinization, and the microstructure generated during thermal processing. Starch gelatinization and subsequent expansion determine parameters such as true density (RD), bulk density, and porosity, which directly influence milk absorption and the perception of crispness [4]. Moreover, thermal processing may alter solid solubility and the availability of phenolic compounds, either through partial degradation or through the release of cell wall–bound fractions [1]. In matrices enriched with tuber‐derived ingredients, the presence of dietary fiber and structural polysaccharides promotes the formation of more compact and mechanically resistant structures, thereby affecting liquid absorption kinetics and stability during consumption [8]. Consequently, the combination of blue corn flour (BCF) and PSPF may constitute a hybrid system capable of balancing expansion, structural integrity, and antioxidant potential.

Beyond structural properties, the antioxidant activity of the final product depends both on the initial content of bioactive compounds and on their stability during thermal processing [9]. Moderate reductions in anthocyanins and flavonoids following heat treatments have been reported, with higher retention observed when acylated anthocyanins predominate, as is the case in purple sweet potato [5]. Within this framework, the strategic combination of BCF and PSPF represents an opportunity to develop flakes with an enhanced antioxidant profile and modulated structural properties while maintaining adequate sensory acceptability. However, the combined effect of both raw materials on microstructure, milk absorption kinetics, bioactive compound stability, and antioxidant activity has not been extensively documented in flake‐type systems. Although several studies have evaluated pigmented cereals or sweet potato independently, limited information is available regarding the combined use of blue corn and purple sweet potato in ready‐to‐eat flake products and the associated effects on functional, antioxidant, physicochemical, and sensory properties. Therefore, the objective of the present study was to evaluate the effect of different proportions of BCF and PSPF on the physicochemical, structural, technofunctional, antioxidant, and sensory properties of ready‐to‐eat flakes.

## 2. Materials and Methods

The raw materials were obtained from the community of Buenos Aires El Apompo, Tuxtepec, Oaxaca, Mexico. Blue corn kernels (*Z. mays* L.) were subjected to a cleaning process and subsequently dried at 60°C for 24 h. The dried kernels were then milled and sieved through a No. 40 mesh sieve (0.42 mm aperture) to obtain BCF. Purple sweet potato (*I. batatas* L.) was washed, sliced into approximately 1 mm thick pieces, and immersed in a 0.5% sodium bisulfite solution (NaHSO_3_) for 30 min. The slices were then held in distilled water for 1 h, drained, and dried at 50°C for 3 h, following the procedure described by Yang et al. [9]. The dried material was milled to obtain a particle size of 0.42 mm aperture (No. 40 mesh).

### 2.1. Preparation of Flakes

A total of 50 g of the previously formulated and homogenized flour blend (Flakes were produced using 100% BCF, 100% PSPF, and blended formulations at ratios of 75/25, 50/50, and 25/75 (BCF/PSPF), respectively), according to the experimental design, were evenly distributed in a nonstick pan. Subsequently, 20 mL of water was atomized onto the surface, and the pan was placed on a preheated heating plate at 200°C for 5 min.

The formed product was carefully detached, subjected to additional drying at 60°C for 90 min, and subsequently broken into flakes. The resulting flakes were stored at 4°C until further analysis.

### 2.2. Physicochemical and Sensory Characterization

The chemical composition was determined according to the official AOAC [10] methods: moisture content by oven drying (Method 934.01), ash by dry incineration (Method 942.05), lipids by petroleum ether extraction (Method 954.02), and protein content by the Kjeldahl method using a nitrogen conversion factor of N × 6.25 (Method 2001.11). Carbohydrate content was estimated by difference. Total energy value was calculated as reported by Hernández‐Santos et al. [11].

WAC and water solubility capacity (WSC) were determined according to Hernández‐Santos et al. [11], using Equations (1) and (2). Milk absorption capacity (MAC) and milk solubility capacity (MSC) were evaluated using the same procedure, substituting water with pasteurized whole milk (protein 3.1%, total fat 3.3%, sugars 4.8%, sodium 46 mg, calcium 116 mg, vitamin A 66.4 *μ*g retinol equivalents, and vitamin D 0.5 *μ*g). Milk absorption kinetics were assessed following the MAC methodology at soaking times of 30 s, 1.0, 1.5, and 2.0 min.

(1) WAC or MAC=Gel weight gSample weight g

(2) WSC or MSC=Solids solubles weight gSample weight g×100

Color measurements were performed using a Hunter tristimulus colorimeter (UltraScan VIS, Model USVIS1347, Hunter Associates Laboratory, Inc., Reston, Virginia, United States). The L*, a*, and b* parameters were used to calculate chroma (C*), hue angle (h°), and total color difference (*Δ*E) [12], using Equations (3), (4), and (5), respectively. The pH was measured in a dispersion of 1 g of sample in 10 mL of distilled water at 25°C.

(3) C∗=a∗2+b∗2

(4) ho=tan−1b∗a∗

(5) ΔE=Ls−L2+as−a2+bs+b212/

RD and bulk density (BD) were determined according to Navarro‐Cortez et al. [13], and porosity (Po) was calculated following Özer et al. [14], using Equations (6), ((7) and (8), respectively.

(6) BD=Weight of flour in container g Volume of containercm3

(7) RD=Weight of seed dipped in water g Volume of water displacedcm3

(8) Po=1−BD RD×100

Water activity (Aw) was measured at 25°C using an AquaLab meter (Model 3TE; Decagon Devices, Inc., Pullman, Washington, United States). Breaking force was evaluated as the maximum resistance to fracture under compression, according to Rodríguez‐Miranda et al. [15], using a TA‐XT2 texture analyzer (Texture Technologies Corp., Scarsdale, NY/Stable Micro Systems, Haslemere, Surrey, United Kingdom) equipped with a Warner–Bratzler blade. Texture results were expressed as the mean of 15 determinations in Newtons (N).

The sensory evaluation was conducted in accordance with institutional ethical guidelines for research involving human participants. All panelists were informed about the study objectives and the ingredients used in the formulations prior to participation, and written informed consent was obtained from all participants. The panel consisted of 100 untrained consumers over 18 years of age who voluntarily participated and reported no known allergies or intolerances to corn, sweet potato, or milk‐derived products. All samples were coded with random three‐digit numbers to preserve participant anonymity, and the study complied with the ethical principles established by the Declaration of Helsinki. Samples were presented in random order, and participants evaluated color, aroma, texture, flavor, and overall acceptability using a 9‐point hedonic scale (1 = *dislike extremely*; 9 = *like extremely*), following Gan et al. [16].

### 2.3. Antioxidant Capacity

#### 2.3.1. Extraction Procedure

Extracts were obtained from 3.0 g of ground powder using 30 mL of solvent (80% ethanol). The mixture was subjected to ultrasonic treatment in an ultrasonic bath for 3 min at a frequency of 80 Hz and 100% power. Subsequently, the samples were centrifuged at 3000 rpm for 20 min. The supernatant obtained after centrifugation was used for the determination of total phenolic compounds as well as for the analysis of total flavonoids, DPPH radical scavenging activity, and ABTS radical scavenging activity, following the method described by Ma et al. [17].

#### 2.3.2. Determination of Total Polyphenols (Folin–Ciocalteu Assay)

Total phenolic content was determined using the Folin–Ciocalteu method, with gallic acid as the standard. Standard solutions at concentrations of 20, 40, 60, 80, and 100 ppm were prepared to construct the calibration curve (R^2^ = 0.999). All determinations were performed in triplicate, and results were expressed as milligrams of gallic acid equivalents (mg GAE) per gram of sample, according to Rodríguez‐Miranda et al. [18].

#### 2.3.3. Total Flavonoid Content (TFC)

TFC was determined according to the procedure described by Rodríguez‐Miranda et al. [18]. A calibration curve was constructed using catechin as the standard at five concentrations (20–100 *μ*g/mL). Results were expressed as milligrams of catechin equivalents (mg CE) per gram of sample.

#### 2.3.4. Determination of ABTS•^+^ Antioxidant Activity

Antioxidant activity was determined using the ABTS•^+^ radical cation decolorization assay following the methodology described by Re et al. [19]. Briefly, the reduction of the ABTS•^+^ radical by antioxidant compounds present in the samples was monitored spectrophotometrically. The antioxidant capacity of the extracts was expressed as IC_50_ values (mg mL^−1^), defined as the concentration of sample required to inhibit 50% of the ABTS•^+^ radical. Lower IC_50_ values indicate greater antioxidant capacity, using Equation (9).

(9) Inhibition %=Αc−ΑsΑc×100

where Ac is the absorbance of the control, and As is the absorbance of the sample. The percentage of inhibition is used to find the concentration at which 50% of the radical is trapped.

#### 2.3.5. Determination of DPPH•^+^ Antioxidant Activity

Antioxidant activity was evaluated using the 2,2‐diphenyl‐1‐picrylhydrazyl (DPPH•) radical scavenging assay. A 0.1‐mM DPPH solution was prepared in methanol, and a calibration curve was constructed using concentrations of 0.02, 0.04, 0.06, 0.08, and 0.10 mM, with methanol as the blank.

Antioxidant activity was expressed as IC_50_ (mg mL^−1^), defined as the concentration required to inhibit 50% of the DPPH radical [2], using Equation (10).

(10) Inhibition %=Αc−ΑsΑc×100

where Ac is the absorbance of the control, and As is the absorbance of the sample. The percentage of inhibition is used to find the concentration at which 50% of the radical is trapped.

#### 2.3.6. Determination of *β*‐Carotene Content

*β*‐Carotene content was quantified by UV–Vis spectrophotometry using a Cary 60 spectrophotometer (Agilent Technologies, Inc., United States), following the procedure described by Ying et al. [20]. The absorbance of the hexane extract was measured at 450 nm [21], using Equation (11).

(11) β−Carotene Content=A450×V×Dε×L×m

where A_450_ is the absorbance measured at 450 nm, V is the volume of the hexane extract (mL), D is the dilution factor, *ε* is the specific extinction coefficient of *β*‐carotene in hexane (2592 L·g^−1^·cm^−1^), L is the path length of the cuvette (cm), and m is the dry weight of the sample (g). Results were expressed as milligrams of *β*‐carotene per gram of sample on a dry basis (mg *β*‐carotene/g dw).

#### 2.3.7. Determination of Total Anthocyanin Content

Total anthocyanin content was determined using the pH differential method, which quantifies monomeric anthocyanins based on spectrophotometric measurements at pH 1.0 and pH 4.5, following Cuevas‐Montilla et al. [22]. Absorbance was measured at 510 and 700 nm using a UV–Vis spectrophotometer against a reagent blank. The corrected absorbance (A) was calculated using Equation (12):

(12) A=Amaxvis−A700pH1.0−Amaxvis−A700pH4.5.

Monomeric anthocyanin content was calculated as cyanidin‐3‐glucoside equivalents using Equation (2):

(13) Monomeric anthocyanins mg100 g=A×MW×DF×100ε×ι

where A is the corrected absorbance, MW is the molecular weight of cyanidin‐3‐glucoside (449.2 g/mol), DF is the dilution factor, *ε* is the molar absorptivity (26,900 L·mol−1·cm−1), and l is the path length of the cuvette (1 cm).

### 2.4. Statistical Analyses

All formulations were prepared in three independent production batches, and analytical determinations were performed in triplicate for each batch. Results are reported as mean ± standard deviation. A completely randomized design was used to evaluate five formulations based on the proportion of BCF and PSPF: 100/0, 75/25, 50/50, 25/75, and 0/100 (w/w). Data were analyzed using analysis of variance (ANOVA), and mean comparisons were performed using Fisher′s Least Significant Difference (LSD) test at a significance level of *p* < 0.05. Statistical analyses were conducted using Statistica software (Version 8.0; StatSoft, Inc., Tulsa, Oklahoma, United States).

## 3. Results and Discussion

### 3.1. Physicochemical Characterization

Moisture content in the flakes ranged from 7.89 to 8.83 g/100 g (Table 1), values below the maximum recommended limit of 15 g/100 g required to ensure storage stability in ready‐to‐eat cereals [11]. Flakes formulated with 100% BCF showed significantly higher ash, protein, and lipid contents, reflecting the intrinsic composition of BCF. Among the blends, the 75BCF/25PSPF formulation exhibited the highest protein and lipid levels, whereas flakes produced with 100% PSPF showed the highest fiber content. Fiber increased progressively with the proportion of PSPF, and the 25BCF/75PSPF formulation presented the highest fiber and carbohydrate contents.

**Table 1:** Physicochemical properties of the flakes blue corn flour (BCF)/purple sweet potato flour (PSPF) blends.

Parameter	Blends (%)				
	BCF	75BCF/25PSPF	50BCF/50PSPF	25BCF/75PSPF	PSPF
**Chemical composition (g/100 g)**					
Moisture	8.57 ± 0.10^c^	8.83 ± 0.19^c^	7.04 ± 0.13^a^	8.54 ± 0.22^c^	7.89 ± 0.05^b^
Ash	1.55 ± 0.06^b^	1.23 ± 0.06^b^	1.22 ± 0.04^c^	1.14 ± 0.03^b^	1.01 ± 0.05^a^
Protein	5.63 ± 0.07^d^	5.14 ± 0.02^c^	3.59 ± 0.10^b^	3.53 ± 0.05^b^	3.25 ± 0.04^a^
Fat	3.82 ± 0.01^e^	3.51 ± 0.04^d^	2.27 ± 0.10^c^	1.53 ± 0.05^b^	0.81 ± 0.04^a^
Fiber	3.03 ± 0.13^a^	5.80 ± 0.09^b^	5.86 ± 0.17^b^	6.00 ± 0.28^b^	8.72 ± 0.31^c^
Carbohydrates	86.39 ± 0.06^b^	84.33 ± 0.10^a^	87.16 ± 0.25^c^	87.39 ± 0.25^c^	86.20 ± 0.38^b^
Total energy (kJ/100 g)	1680.51 ± 1.89^e^	1626.52 ± 1.61^d^	1597.10 ± 5.21^c^	1576.12 ± 3.22^b^	1524.61 ± 6.37^a^
**Physicochemical**					
Luminosity (L*)	69.47 ± 0.46^c^	62.26 ± 0.07^b^	58.94 ± 0.29^ab^	57.32 ± 0.23ª	56.15 ± 2.97ª
Chromaticity +red to −green (a*)	6.31 ± 0.22ª	10.26 ± 0.50^b^	12.43 ± 0.36^c^	13.67 ± 0.25^d^	12.80 ± 0.41^cd^
Chromaticity +yellow to −blue (b*)	4.42 ± 0.07^d^	1.70 ± 0.43^c^	0.83 ± 0.19^b^	0.21 ± 0.01ª	1.80 ± 0.08^c^
Chromaticity (C*)	29.67 ± 1.69ª	54.26 ± 5.99^b^	77.65 ± 4.62^c^	93.52 ± 3.34^d^	83.55 ± 5.16^cd^
Hue angle (*h°*)	35.03 ± 0.54^d^	9.34 ± 1.85^c^	3.82 ± 0.74^b^	0.88 ± 0.06ª	8.00 ± 0.57^c^
Total color difference (*Δ*E)	30.62 ± 0.40^a^	38.57 ± 0.20^b^	42.37 ± 0.17^c^	44.29 ± 0.30^c^	45.15 ± 2.71^c^
pH	6.01 ± 0.03^b^	5.90 ± 0.03^ab^	5.90 ± 0.09^ab^	5.88 ± 0.02ª	5.83 ± 0.04^a^
Real density (RD g/cm^3^)	0.87 ± 0.01ª	1.01 ± 0.01^d^	1.22 ± 0.00^c^	1.52 ± 0.02^b^	1.52 ± 0.02^d^
Apparent density (AD g/cm^3^)	0.20 ± 0.01^a^	0.21 ± 0.00^ab^	0.27 ± 0.01^c^	0.27 ± 0.01^c^	0.23 ± 0.01^b^
Porosity	0.76 ± 0.00^b^	0.73 ± 0.01^a^	0.78 ± 0.01^c^	0.85 ± 0.00^d^	0.83 ± 0.00^e^
Water activity (A_w_)	0.59 ± 0.03^b^	0.38 ± 0.01ª	0.38 ± 0.00^a^	0.39 ± 0.00^a^	0.39 ± 0.00^a^
Breaking force (N)	2.42 ± 0.99^b^	4.22 ± 2.33^c^	3.12 ± 0.92^b^	3.16 ± 1.16^bc^	3.60 ± 1.20^bc^
Thickness (mm)	0.61 ± 0.01ª	0.58 ± 0.00^a^	0.54 ± 0.01ª	0.48 ± 0.01ª	0.91 ± 1.15ª

*Note:* The results are the average of three determinations ± standard deviation. Values are expressed on a dry basis, except moisture. The mean values in the same row followed by a different superscript are significantly (*p* < 0.05) different. 75BCF/25PSPF = 75% of BCF and 25% of PSPF; 50BCF/50PSPF = 50% of BCF and 50% of PSPF; 25BCF/75PSPF = 25% of BCF and 75% of PSPF; *Δ*E = total color difference.

These differences are mainly attributed to the compositional contribution of each ingredient and dilution effects within the blends. Although starch–lipid interactions such as amylose–lipid complex formation may occur during processing and affect lipid availability and matrix organization, the fat content of the flakes was primarily determined by the native composition of the raw materials.

Protein content was lower than that reported by Al‐Okbi et al. [23] for corn flakes (10.27 g/100 g) and by Fasuan et al. [24] for flakes formulated with amaranth, soy protein, and modified starch (12.9–40 g/100 g). However, it was within the range described by Fitriani et al. [25] for rice–banana flakes (2.4–4.4 g/100 g). Lipid content was consistent with values reported in these studies (1.8–7.46 g/100 g), whereas fiber levels were comparable with those described by Fasuan et al. [24] (3.34–6 g/100 g). The highest energy values (*p* < 0.05) corresponded to formulations with greater proportions of BCF, which can be attributed to their higher lipid content, the macronutrient contributing most to caloric value. The energy values obtained were lower than those reported by Hu et al. [26] for oat flakes (1757.20–1783.83 kJ/100 g) but fell within the range reported for corn flakes by Al‐Okbi et al. [23] (1570.22–1680.29 kJ/100 g).

Color parameters differed significantly among formulations. The L* value increased with the proportion of BCF, with the 75BCF/25PSPF blend presenting the highest lightness. The a* parameter was higher in PSPF‐rich formulations, although all samples remained within the red hue quadrant. In contrast, the b* parameter was higher in BCF and decreased significantly (*p* < 0.05) as PSPF proportion increased. All formulations showed yellow tonalities. Chroma (C*) and total color difference (*Δ*E) were highest in the 25BCF/75PSPF formulation and decreased significantly as the proportion of BCF increased, whereas the highest hue angle (h°) was observed in the 75BCF/25PSPF blend. These variations are mainly attributed to the presence of anthocyanins and other pigments in both raw materials and to the dilution effect between them [27, 28].

The highest pH value was recorded in BCF; however, no significant differences (*p* > 0.05) were observed between blends and PSPF. All formulations showed slightly acidic pH values (5.83–6.01), consistent with those reported for pigmented corn flours and related cereal products [2, 3]. The determination of pH is relevant because it influences pigment stability, antioxidant retention, and the functional behavior of starch‐based matrices during processing and storage. In the present study, the absence of significant differences among formulations suggests that blending BCF and PSPF did not markedly alter the acid–base balance of the system, likely due to the similar contribution of organic acids and phenolic compounds naturally present in both pigmented raw materials [29, 30].

RD increased significantly (*p* < 0.05) as the proportion of PSPF increased, from 0.87 g/cm^3^ in flakes produced exclusively with BCF to 1.52 g/cm^3^ in the 25BCF/75PSPF formulation. This behavior reflects greater solid‐phase compaction due to the higher content of dietary fiber, structural polysaccharides, and tuber starch in purple sweet potato. Similar trends have been reported in products formulated with root or tuber flours, which tend to form denser matrices because their starches show lower expansion capacity and fiber interferes with steam bubble formation during thermal processing [8, 31].

In contrast, flakes containing higher proportions of BCF exhibited lower RD values due to the high expansion capacity of cereal starch, which promotes the formation of cellular structures with thin walls and greater internal air volume [4]. This behavior can be explained by structural differences between cereal and tuber starches. Corn starch typically presents smaller granule size and relatively higher amylose content compared with sweet potato starch, favoring stronger melt elasticity and improved bubble stabilization during thermal processing, which enhances expansion and reduces product density [32, 33]. In contrast, sweet potato starch is characterized by larger granules, higher swelling power, and lower gelatinization temperature, as well as a matrix rich in nonstarch polysaccharides that can limit bubble growth and promote the formation of more compact structures [34]. Additionally, the presence of dietary fiber in PSPF further restricts expansion by interrupting the continuity of the starch matrix and weakening cell wall formation, resulting in denser structures and higher RD values. Therefore, the observed RD differences between blends are associated not only with dilution effects but also with intrinsic physicochemical differences between cereal and tuber starch systems that govern structure formation during flake processing.

Apparent density (AD) ranged from 0.20 to 0.27 g/cm^3^ and was significantly lower than RD in all formulations, confirming the development of highly aerated structures. Formulations with higher BCF content showed the lowest AD values, indicating greater expansion and reduced structural compaction, a desirable characteristic in ready‐to‐eat cereals. Similar behavior has been reported in extruded corn‐based products, where thermal expansion produces lightweight matrices with low AD [1].

As PSPF content increased, AD slightly increased, suggesting reduced volumetric expansion and stronger matrix cohesion. This behavior is associated not only with the reinforcing effect of dietary fiber and phenolic compounds but also with differences in starch characteristics between cereal and tuber sources [5]. Although starch is the dominant structural component in the formulations, partial replacement of cereal starch with sweet potato starch modifies expansion behavior due to its larger granule size, higher swelling capacity, and lower gelatinization temperature, which tend to limit bubble growth and promote the formation of more compact structures [32, 34]. In addition, the presence of dietary fiber and nonstarch polysaccharides in PSPF disrupts the continuity of the starch matrix, further reducing expansion and contributing to higher AD values.

Porosity values were high in all formulations (0.73–0.85), confirming the development of porous structures typical of breakfast cereals. PSPF‐rich formulations showed slightly higher porosity values, indicating the formation of smaller and more uniformly distributed microcells that increased the volumetric air fraction without proportionally reducing solid density. Similar microstructures have been reported in products formulated with tubers and legumes [14, 35].

Aw ranged from 0.38 to 0.59. Flakes produced with 100% BCF showed the highest value (0.59), whereas formulations containing PSPF exhibited significantly lower values (0.38–0.39; *p* < 0.05). The decrease in Aw with PSPF incorporation suggests reduced water availability due to interactions with dietary fiber, nonstarch polysaccharides, and phenolic compounds present in purple sweet potato. Similar Aw values (0.55–0.65) have been reported for cereal‐based systems with low fiber content [1], whereas lower Aw values (< 0.45) have been observed in functional products containing purple sweet potato [5, 6]. All values obtained in this study were below 0.60, indicating adequate microbiological stability for ready‐to‐eat cereals.

Breaking force ranged from 2.42 to 4.22 N (*p* < 0.05). Flakes produced with 100% BCF exhibited the lowest value, whereas the 75BCF/25PSPF formulation presented the highest mechanical resistance. Intermediate PSPF levels (50%–100%) showed moderate values (≈3.12–3.60 N), suggesting reinforcement of the matrix by the incorporation of purple sweet potato. The lower fracture resistance in BCF‐rich flakes is related to the high expansion capacity of cereal starch, which produces highly aerated structures with thin cell walls. Similar values (2–3 N) have been reported for expanded corn‐based cereals [1]. In contrast, higher fiber and structural polysaccharide contents in PSPF strengthen the matrix and increase fracture resistance, as reported in root‐ and tuber‐based products [8, 31]. The maximum value observed in the 75BCF/25PSPF formulation suggests a synergistic balance between expansion and structural reinforcement, previously described in cereal–tuber systems [35].

Flake thickness ranged from 0.48 to 0.61 mm in formulations containing BCF and their blends, whereas 100% PSPF flakes exhibited greater thickness and variability. Thickness slightly decreased as PSPF increased up to 75%, suggesting greater structural compaction. However, thickness did not correlate directly with fracture force, indicating that internal microstructure and pore architecture have a stronger influence on mechanical resistance than external geometry, as previously reported for extruded cereals [4, 14]. Overall, formulations containing intermediate PSPF levels (50%–75%) showed a favorable balance between expansion, mechanical resistance, and structural stability, suggesting promising technological and functional properties for the development of pigmented cereal flakes.

### 3.2. Functional Properties

#### 3.2.1. WAC

**Figure 1:**
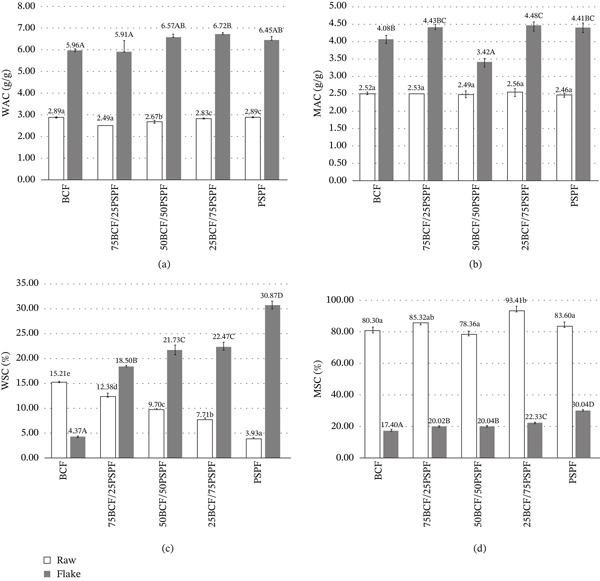
Effect on the: (a) water absorption capacity (WAC), (b) milk absorption capacity (MAC), (c) water solubility capacity (WSC), and (d) milk solubility capacity (MSC) of the raw samples and flakes of the different blends of blue corn flour (BCF)/purple sweet potato flour (PSPF). Lowercase letters indicate a significant difference (*p* < 0.05) in the raw samples, and capital letters indicate a significant difference (*p* < 0.05) in the flakes.

Figure 1a shows that WAC was significantly higher (*p* < 0.05) in flakes than in raw samples for all formulations, indicating structural changes caused by thermal processing. This increase is mainly attributed to partial starch gelatinization, granule disruption, and greater exposure of hydrophilic groups, which enhance water retention. Similar behavior has been widely reported in breakfast cereals and extruded products, where heat treatment increases hydration capacity [1, 4].

WAC also increased progressively with the proportion of PSPF in both raw samples and flakes. This behavior is mainly associated with the higher content of dietary fiber present in PSPF, which includes nonstarch polysaccharides such as cellulose, hemicelluloses, and pectic substances, as well as resistant starch fractions that contribute to water retention through hydrogen bonding and matrix swelling mechanisms. These hydrophilic components increase the availability of hydroxyl groups capable of interacting with water molecules and promote the formation of more hydrated structures within the starch‐based matrix [34, 36]. In addition, the incorporation of sweet potato flour modifies the continuity of the cereal starch network, increasing porosity and enhancing WAC in both raw blends and processed flakes [36]. The incorporation of tuber flours has been reported to increase water absorption because fiber immobilizes water within the structural matrix [8, 31]. Conversely, formulations with higher proportions of BCF showed lower WAC values, a behavior typical of cereal‐based matrices where hydration mainly depends on starch gelatinization and expansion. Similar trends have been reported for pigmented corn flakes and cereal snacks [14].

#### 3.2.2. MAC

MAC values (Figure 1b) were significantly higher (*p* < 0.05) in flakes than in raw samples, demonstrating that thermal processing improves the interaction of the product with complex liquid systems such as milk. However, MAC values were consistently lower than those observed for WAC. This difference can be explained by the higher viscosity and compositional complexity of milk, which contains proteins, lipids, and lactose that limit liquid penetration into porous structures. Similar results have been described for ready‐to‐eat cereals intended for consumption with milk [6].

Increasing the proportion of PSPF slightly increased MAC values, suggesting that dietary fiber and structural polysaccharides from purple sweet potato enhance milk retention through the formation of a more efficient capillary network. Nevertheless, the increase remained moderate, which is technologically desirable because excessive liquid absorption can accelerate the loss of crispness during consumption. Intermediate absorption values have been associated with improved performance of cereal products in cereal–milk systems [5].

#### 3.2.3. WSC

WSC was significantly higher (*p* < 0.05) in flakes than in raw samples across all formulations (Figure 1c), confirming the effect of thermal processing on component solubilization. This increase is related to starch gelatinization and partial granule disruption, processes that promote the release of amylose and other soluble fractions into the aqueous phase. Such behavior is characteristic of thermally processed cereals and extruded products [1, 4].

A progressive increase in WSC was observed as the proportion of BCF increased. This trend may be attributed to the greater susceptibility of cereal starch to gelatinization and amylose leaching compared with tuber starches. In corn‐based products, higher WSC values are commonly associated with greater starch degradation during processing [14]. In contrast, formulations containing higher proportions of PSPF showed lower WSC values.

This behavior can be explained not only by the higher dietary fiber content of PSPF, which includes nonstarch polysaccharides capable of limiting starch solubilization and retaining solids within the matrix, but also by structural modifications occurring in the starch fraction during processing [8]. In particular, the formation of resistant starch through amylose reassociation and retrogradation during cooling contributes to reduced solubility by promoting more ordered crystalline regions within the matrix. Additionally, the hydrophilic hydroxyl groups of starch favor water binding, whereas hydrophobic interactions between reassociated amylose chains restrict molecular dispersion, further decreasing solubilized material in the aqueous phase. The presence of phenolic compounds and fiber components in PSPF may also interact with starch chains and reinforce matrix integrity, limiting polymer leaching. Similar behavior has been reported in tuber‐based systems where resistant starch formation and starch retrogradation reduce solubility even after thermal processing [34, 36, 37].

#### 3.2.4. MSC

MSC values (Figure 1d) were lower than WSC in both raw samples and flakes. This behavior is related to the compositional complexity of milk, whose proteins and lipids interact with food components and reduce the release of soluble solids. Similar observations have been reported for ready‐to‐eat cereals consumed with milk [6].

Although MSC increased after thermal processing, indicating that flake production promotes the release of soluble fractions even in complex media, formulations with higher BCF content exhibited the highest MSC values, whereas increasing PSPF reduced solubility. This reduction may be associated with dietary fiber and phenolic compounds from purple sweet potato, which interact with milk proteins and limit the diffusion of soluble solids. From a technological perspective, moderate solubility values are desirable because they prevent excessive milk turbidity and rapid loss of crispness. Intermediate BCF/PSPF formulations showed an adequate balance between solubility and stability, similar to commercial breakfast cereals [5, 14].

#### 3.2.5. Milk Absorption Kinetics

**Figure 2:**
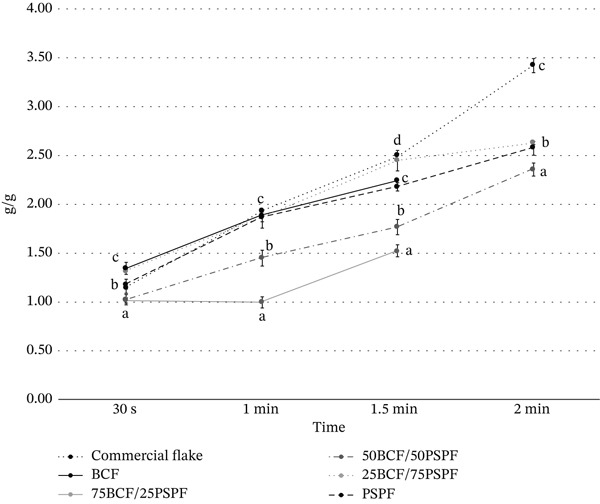
Milk absorption kinetics of the flakes of the different blends of blue corn flour (BCF) and purple sweet potato flour (PSPF). Different letters at the same time indicate significant difference (*p* < 0.05).

Milk absorption kinetics (Figure 2) is an important functional parameter in ready‐to‐eat cereals because it influences rehydration behavior and crispness during consumption. All BCF/PSPF formulations showed rapid absorption during the first 30–60 s followed by a stabilization phase, a typical pattern for flake‐type cereals.

The commercial cereal showed faster and more pronounced absorption, reaching high values within short time intervals. This behavior is associated with its highly expanded structure, low density, and large porosity, characteristics commonly observed in industrial cereals based on refined starch [1, 4]. Flakes produced with 100% BCF exhibited relatively rapid initial absorption, although less pronounced than the commercial cereal, suggesting an expanded but mechanically stronger structure.

In contrast, formulations containing higher proportions of PSPF showed slower and more controlled absorption kinetics (*p* < 0.05), particularly during the initial stages. This behavior is related to their higher density, greater fracture resistance, and more compact microstructure, as well as the higher fiber content of purple sweet potato, which acts as a barrier to fluid penetration. Similar gradual absorption patterns have been reported in tuber‐ and fiber‐enriched matrices [8, 31].

Intermediate BCF/PSPF formulations exhibited a balanced kinetic profile, combining adequate rehydration with slower absorption compared with the commercial cereal. This behavior is technologically advantageous because it allows the product to maintain crispness for longer periods without compromising sensory acceptability [6, 14]. Overall, the incorporation of PSPF improves both the functional performance and structural stability of the flakes during consumption.

### 3.3. Bioactive Compounds and Antioxidant Capacity

#### 3.3.1. Anthocyanins

In raw samples, monomeric anthocyanin content increased with the proportion of PSPF, ranging from 86.13 mg/100 g in BCF to 120.37 mg/100 g in PSPF. Intermediate values were observed for the blends 75BCF/25PSPF (108.03 mg/100 g), 50BCF/50PSPF (109.69 mg/100 g), and 25BCF/75PSPF (114.85 mg/100 g) (*p* < 0.05, Table 2). This trend reflects the higher anthocyanin concentration in purple sweet potato, characterized mainly by acylated anthocyanins, together with the contribution of anthocyanin derivatives typical of pigmented corn.

**Table 2:** Content of anthocyanins, *β*‐carotene, flavonoids, and polyphenols in the raw samples and flakes of the different blends of blue corn flour (BCF)/purple sweet potato flour (PSPF).

Parameter	Blends (%)				
	BCF	75BCF/25PSPF	50BCF/50PSPF	25BCF/75PSPF	PSPF
Monomeric anthocyanins (mg/100 g)					
Raw	86.13 ± 0.19^a^	108.03 ± 0.12^b^	109.69 ± 1.01^c^	114.85 ± 0.29^d^	120.37 ± 0.03^e^
Flake	82.21 ± 0.31^e^	79.60 ± 0.18^d^	63.73 ± 0.37^b^	69.50 ± 0.19^c^	57.35 ± 0.06^a^
Change (%)	4.56	26.32	41.9	39.49	52.35
*β*‐carotene content (mg *β*‐C/g)					
Raw	17.43 ± 0.02^d^	17.22 ± 0.08^cd^	16.60 ± 0.42^c^	15.50 ± 0.33^b^	11.95 ± 0.08^a^
Flake	7.55 ± 0.02^e^	6.49 ± 0.01^d^	5.61 ± 0.04^c^	4.95 ± 0.01^b^	4.08 ± 0.01^a^
Change (%)	36.78	58.09	66.21	71.25	76.62
Total flavonoid content (mg Cat E/g)					
Raw	35.35 ± 0.19^a^	37.48 ± 0.28^b^	39.29 ± 0.29^c^	38.52 ± 0.49^bc^	42.95 ± 0.82^d^
Flake	33.73 ± 0.82^a^	35.08 ± 0.28^b^	36.18 ± 0.29^b^	38.98 ± 0.49^c^	39.61 ± 0.19^c^
Change (%)	3.11	0.84	1.12	‐0.18	‐1.69
Total polyphenol content (mg GA E/g)					
Raw	54.09 ± 0.33^e^	52.38 ± 0.73^d^	44.72 ± 0.24^c^	41.73 ± 0.07^b^	38.03 ± 0.01^a^
Flake	99.62 ± 0.60^c^	97.57 ± 0.93^c^	100.02 ± 0.26^c^	88.77 ± 0.78^a^	92.95 ± 1.82^b^
Change (%)	84.25	95.19	124.03	83.8	144.39

*Note:* The results are the average of three determinations ± standard deviation. The mean values in the same row followed by a different superscript are significantly (*p* < 0.05) different. 75BCF/25PSPF = 75% of BCF and 25% of PSPF; 50BCF/50PSPF = 50% of BCF and 50% of PSPF; 25BCF/75PSPF = 25% of BCF and 75% of PSPF.

After processing into ready‐to‐eat flakes, all formulations showed a significant reduction in monomeric anthocyanins. Relative losses ranged from 4.56% in BCF to 52.35% in PSPF (Table 2). This reduction is associated with the analytical principle of the pH differential method, which quantifies only monomeric anthocyanins; therefore, heat‐induced transformations such as polymerization, copigmentation, condensation with Maillard reaction products, or degradation into phenolic derivatives are reflected as decreases in monomeric content. These reactions are common in cereal‐based systems during thermal processing, where the presence of reducing sugars, proteins, oxygen, and moisture promotes pigment transformation. Although PSPF exhibited the highest initial anthocyanin content, it also presented the greatest reduction after processing. This behavior may be attributed to the larger initial pigment pool available for degradation or structural transformation, as well as to differences in microenvironmental conditions such as pH, Aw, and matrix structure during heating. In contrast, the 100% BCF formulation retained most of its monomeric anthocyanins, suggesting a possible protective matrix effect related to interactions with starch or proteins that may limit pigment degradation.

Reported reductions during cereal and snack processing commonly range from moderate to high depending on thermal severity [5, 6, 38, 39]. In the present study, losses between 4.56% and 52.35% fall within these ranges. Notably, intermediate blends (25BCF/75PSPF and 50BCF/50PSPF) retained higher final monomeric anthocyanin levels (≈63.73–69.50 mg/100 g) than PSPF alone (≈57.35 mg/100 g), suggesting a potential matrix effect of blue corn that may contribute to improved anthocyanin retention during processing.

#### 3.3.2. *β*‐Carotene

*β*‐Carotene content (mg *β*‐C/g) in the raw blends showed significant differences (*p* < 0.05) among formulations (*p* < 0.05), ranging from 11.95 to 17.43 mg *β*‐C/g (Table 2). The highest value was observed in BCF (17.43 mg/g), whereas PSPF presented the lowest (11.95 mg *β*‐C/g). Intermediate concentrations were recorded for the blends (17.22, 16.60, and 15.50 mg *β*‐C/g), reflecting the compositional contribution of each ingredient.

After processing into ready‐to‐eat flakes, *β*‐carotene decreased markedly in all formulations, reaching values between 4.08 and 7.55 mg *β*‐C/g. As observed in raw samples, the highest content was retained in BCF (7.55 mg *β*‐C/g) and the lowest in PSPF (4.08 mg *β*‐C/g). Overall, thermal processing resulted in reductions (change, Table 2) of approximately 36.78%–76.62%, confirming the high susceptibility of carotenoids to degradation during heat treatments. These losses are mainly associated with oxidative degradation and cis–trans isomerization induced by heating, phenomena widely reported in thermally processed cereals and snack products [1]. Additionally, processing steps such as gelatinization, drying, and toasting increase matrix porosity and surface area, facilitating oxygen diffusion and promoting radical‐mediated co‐oxidation reactions.

The magnitude of the losses observed in this study is comparable with values reported for cereal‐based products subjected to extrusion or flaking processes (20%–50%) [40].

The *β*‐carotene concentrations measured in the raw blends (11.95–17.43 mg *β*‐C/g) are also consistent with those previously reported for BCF/PSPF systems, where values between 15.43 and 17.20 mg *β*‐C/g were described depending on formulation and raw material variability [11].

Variations in *β*‐carotene levels between ingredients can be explained by compositional differences among cultivars. In purple sweet potato, the dominant characteristic is anthocyanin accumulation, whereas *β*‐carotene is typically more abundant in orange‐fleshed varieties [41]. Consequently, the reduction observed in flakes reflects the balance between structural development during processing and the inherent thermal sensitivity of carotenoids within the food matrix.

#### 3.3.3. Total Flavonoids

TFC (mg catechin equivalents/g) in the raw blends showed a general increasing trend with the incorporation of PSPF, rising from 35.35 mg Cat E/g in BCF to 42.95 mg Cat E/g in PSPF (*p* < 0.05, Table 2). Intermediate values were observed in the mixed formulations, indicating that PSPF contributed substantially to the flavonoid fraction of the blends.

After processing into ready‐to‐eat flakes, TFC values ranged from 33.73 (BCF) to 39.61 mg Cat E/g (PSPF), maintaining statistical differences among formulations (*p* < 0.05). Transformation into flakes produced only minor changes in most treatments, with small losses between −1.69% and 3.11%. Interestingly, formulations containing higher PSPF levels showed slight negative change (%) values (−0.18 to −1.69%, Table 2), indicating very high retention or an apparent increase in flavonoid content after processing.

This pattern can be explained by the effect of thermal processing on matrix disruption and compound extractability. Colorimetric assays commonly used to determine TFC (e.g., AlCl_3_ complexation) measure extractable flavonoid fractions; therefore, heat treatments that disrupt cell wall structures and enhance solvent penetration may increase the extractability of bound phenolic compounds; however, this effect does not necessarily imply a proportional increase in flavonoid content, since flavonoids represent only a specific subclass within total phenolics and may respond differently to thermal processing depending on their structural stability [7, 42]. Similar behaviors have been reported in extruded or expanded cereal products, where processing can release bound phenolic compounds and increase their extractability despite partial degradation of individual molecules [43].

The results also confirm that PSPF is an important contributor to flavonoid‐related compounds in the blends. The PSPF formulation presented the highest TFC values both in raw and processed samples (42.95 to 39.61 mg Cat E/g), whereas BCF showed the lowest levels (35.35 to 33.73 mg Cat E/g). This trend is consistent with observations in plant‐based matrices where processing modifies microstructure and improves phenolic accessibility [44].

Overall, the BCF/PSPF flakes demonstrated remarkable preservation of flavonoid‐type compounds during processing. Such stability suggests that the fibrous matrix and phenolic–biopolymer interactions may partially protect flavonoids from degradation, supporting the development of ready‐to‐eat cereal products with retained antioxidant potential [45].

#### 3.3.4. Total Polyphenols

The total polyphenol content (TPC) showed significant differences (*p* < 0.05) among both the raw materials and the flake products. Among the raw materials, the highest TPC was observed in BCF (54.09 mg GAE/g), followed by the 75BCF/25PSPF (52.38 mg GAE/g), 50BCF/50PSPF (44.72 mg GAE/g), and 25BCF/75PSPF (41.73 mg GAE/g) blends, whereas PSPF exhibited the lowest value (38.03 mg GAE/g), indicating that blue corn was the primary source of phenolic compounds in the formulations (Table 2).

The higher concentration of polyphenols in BCF can be attributed to the presence of phenolic acids, flavonoids, and anthocyanin‐derived compounds that are characteristic of pigmented maize varieties. Previous studies have demonstrated that blue and purple corn contain higher levels of phenolic compounds than conventional maize due to the accumulation of secondary metabolites associated with phenolic pigments [2]. In fact, Hernández‐Santos et al. [3] reported phenolic compound contents of up to 229.20 mg GAE/100 g in different anatomical structures of pigmented maize, confirming that these materials constitute important natural sources of antioxidants [2].

Following flake production, TPC increased significantly (*p* < 0.05) in all formulations (Table 2). The 50BCF/50PSPF flakes exhibited the highest phenolic content (100.02 mg GAE/g), followed by BCF (99.62 mg GAE/g) and 75BCF/25PSPF (97.57 mg GAE/g), with no significant differences among these samples (*p* > 0.05). In contrast, the 25BCF/75PSPF formulation showed the lowest value (88.77 mg GAE/g), whereas flakes produced exclusively from PSPF displayed an intermediate content (92.95 mg GAE/g). Relative to the corresponding raw materials, TPC increased by 84.25%, 95.19%, 124.03%, 83.805, and 144.39% (change, Table 2), respectively, with particularly pronounced increases observed in the PSPF formulation and the 50BCF/50PSPF blend.

The increase in TPC after processing contrasts with the commonly reported thermal degradation of phenolic compounds and suggests that the applied treatment promoted the release of phenolics previously bound to the food matrix. During heating, starch gelatinization, cell wall disruption, and the cleavage of bonds between phenolic compounds and structural polysaccharides occur, thereby enhancing the extractability of these metabolites [6]. Furthermore, thermal processing may generate reducing compounds derived from Maillard reactions that react with the Folin–Ciocalteu reagent, contributing to the apparent increase in total phenolic content [6].

The behavior observed in PSPF‐containing formulations may also be related to the nature of the phenolic compounds present in purple sweet potato. Several studies have demonstrated that a substantial proportion of sweet potato polyphenols are bound to cell wall components; consequently, thermal treatments enhance their release and analytical recovery [46, 47]. Similarly, Escobar‐Puentes et al. [5] reported that purple‐fleshed sweet potatoes contain considerable amounts of phenolic acids and chlorogenic acid derivatives that can be released during thermal processing, thereby increasing the antioxidant potential of the final product.

Notably, the 50BCF/50PSPF formulation achieved the highest polyphenol content (100.02 mg GAE/g), exceeding even the values observed in flakes produced from a single raw material. This finding suggests a possible synergistic interaction between phenolic compounds derived from blue corn and purple sweet potato. Similar phenomena have been reported in antioxidant‐rich ingredient blends, where the combination of distinct phenolic profiles enhances the stability and extractability of bioactive compounds during processing [5, 6].

These findings are particularly relevant because they demonstrate that the flake manufacturing process not only preserved the phenolic compounds originally present in the raw materials but also significantly increased their availability. This observation is consistent with previous reports on products based on pigmented cereals and tuber crops, in which controlled processing promotes the release of antioxidant compounds and enhances the functional value of the food matrix [46, 47]. Therefore, flakes formulated with BCF and PSPF blends can be considered an important source of phenolic compounds with antioxidant potential, with the 50BCF/50PSPF formulation representing the most promising alternative from a nutraceutical perspective.

#### 3.3.5. Antioxidant Activity (IC₅₀ DPPH• ^+^ and ABTS•^+^)

**Figure 3:**
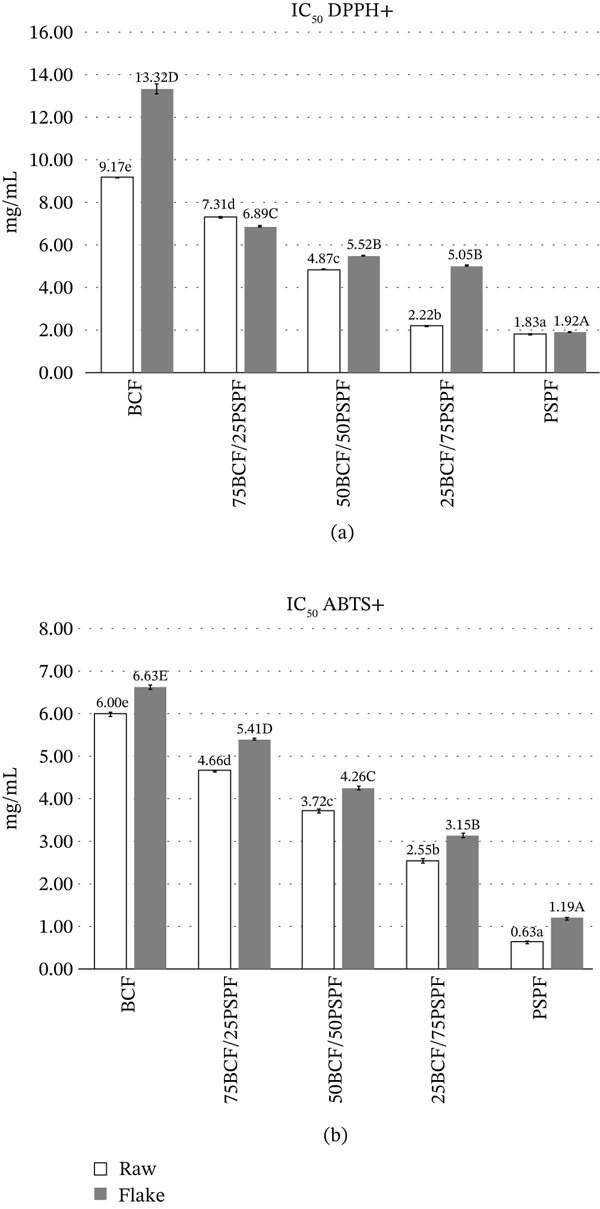
IC_50_ values (a) DPPH+ and (b) ABTS+ of the raw samples and flakes of the different blends of blue corn flour (BCF) and purple sweet potato flour (PSPF). Lowercase letters indicate a significant difference (*p* < 0.05) in the raw samples, and capital letters indicate a significant difference (*p* < 0.05) in the flakes.

In the raw samples, IC_50_ values for the DPPH• radical decreased significantly (*p* < 0.05) as the proportion of PSPF increased (Figure 3a), indicating enhanced radical‐scavenging capacity in PSPF‐enriched formulations. This trend is consistent with the higher levels of monomeric anthocyanins, total flavonoids, and total polyphenols previously reported for PSPF‐rich blends, confirming the strong contribution of these compounds to antioxidant activity.

After processing into ready‐to‐eat flakes, IC_50_ values generally increased compared with the raw samples, indicating a moderate reduction in antioxidant capacity after thermal treatment. Nevertheless, the overall trend remained unchanged: formulations with higher PSPF proportions retained the lowest IC_50_ values, whereas BCF exhibited the highest values. The increase in IC_50_ values observed in flakes can be attributed not only to partial degradation of anthocyanins and carotenoids, oxidation of phenolic compounds, and structural transformations that reduce their reactivity toward the DPPH radical, but also to differences in the phenolic profiles of BCF and PSPF. Blue corn is characterized mainly by anthocyanins such as cyanidin‐3‐glucoside and its derivatives, which are highly sensitive to thermal processing and oxidative conditions, resulting in significant reductions in antioxidant activity after heat treatments. In contrast, purple sweet potato contains predominantly acylated anthocyanins together with phenolic acids and flavonoids that exhibit greater thermal stability but lower radical scavenging efficiency per unit concentration compared with nonacylated anthocyanins. Therefore, variations in IC_50_ among formulations are associated not only with total phenolic losses during processing but also with compositional differences in anthocyanin structure and stability between cereal‐ and tuber‐based matrices. Additionally, interactions between phenolic compounds and starch or dietary fiber components may reduce extractability and accessibility of antioxidants after processing, further contributing to increased IC_50_ values in flakes compared with raw blends [7, 22, 36, 38, 43, 47].

A comparable pattern was observed for ABTS•^+^ scavenging activity (Figure 3b). In the raw blends, IC_50_ values decreased significantly with increasing PSPF proportion, following the gradient: BCF > 75BCF/25PSPF > 50BCF/50PSPF > 25BCF/75PSPF > PSPF. This behavior confirms that PSPF contributed a highly reactive phenolic fraction, particularly anthocyanins and other polyphenols. The ABTS assay is compatible with both hydrophilic and lipophilic antioxidants, allowing a broader evaluation of antioxidant activity in complex cereal–tuber matrices [19]. Following flake processing, IC_50_ values increased across all formulations, indicating a partial reduction in antioxidant activity. However, PSPF‐rich formulations still exhibited the lowest IC_50_ values, demonstrating that phenolic‐rich matrices retained higher antioxidant potential even after thermal treatment. Similar trends have been reported in cereal systems subjected to thermomechanical processing, where phenolic degradation and oxidation occur despite partial retention of total phenolics [43, 47].

Overall, PSPF proportion was the main factor modulating antioxidant capacity. Although processing induced a moderate decline in activity, PSPF‐rich blends particularly 25BCF/75PSPF and PSPF maintained the highest radical‐scavenging capacity after processing, supporting their potential for the development of functional ready‐to‐eat cereals with elevated antioxidant properties [45].

#### 3.3.6. Sensory Attributes

The results in Table 3 show that the BCF/PSPF ratio significantly influenced (*p* < 0.05) several sensory attributes, confirming the important role of formulation in overall product perception. Color scores increased with higher PSPF proportions due to the purple–violet intensity provided by anthocyanins from purple sweet potato, which generally improves consumer preference for naturally pigmented cereal products [5, 6]. However, the 100% PSPF formulation presented darker tones that could reduce preference among some consumers.

**Table 3:** Sensory evaluation of the flakes blue corn flour (BCF)/purple sweet potato flour (PSPF) blends.

Sensory attributes	Blends (%)				
	BCF	75BCF/25PSPF	50BCF/50PSPF	25BCF/75PSPF	PSPF
Color	5.11 ± 2.47^a^	7.10 ± 1.60^ab^	7.56 ± 2.60^b^	8.33 ± 1.94^b^	8.44 ± 2.35^b^
Odor	5.56 ± 1.94^a^	6.80 ± 1.69^ab^	7.22 ± 2.33^ab^	7.78 ± 1.99^b^	8.00 ± 2.40^b^
Texture	5.44 ± 2.51^a^	7.10 ± 1.29^ab^	8.08 ± 1.94^b^	8.67 ± 1.50^b^	8.11 ± 2.47^b^
Taste	4.67 ± 1.54^a^	6.40 ± 1.59^b^	7.33 ± 2.35^bc^	8.44 ± 1.67^c^	8.44 ± 1.81^c^
Overall acceptance	5.19 ± 1.87^a^	6.85 ± 1.40^ab^	7.53 ± 2.11^b^	8.31 ± 1.65^b^	8.25 ± 2.04^b^

*Note:* The results are the average of a hundred observations ± standard deviation. The mean values in the same row followed by a different superscript are significantly (*p* < 0.05) different. 75BCF/25PSPF = 75% of BCF and 25% of PSPF; 50BCF/50PSPF = 50% of BCF and 50% of PSPF; 25BCF/75PSPF = 25% of BCF and 75% of PSPF.

Aroma and flavor scores were highest (*p* < 0.05) in intermediate formulations containing 50% and 75% PSPF, suggesting a complementary sensory effect between the toasted notes of blue corn and the mild sweetness of purple sweet potato. Similar results have been reported in cereal‐based snacks where balanced ingredient blends improve sensory acceptability [14]. Texture also differed significantly among formulations (*p* < 0.05). Samples with higher BCF content were perceived as crispier but softened more rapidly in milk, whereas higher PSPF formulations showed greater firmness and structural stability. Intermediate blends achieved the best texture balance, consistent with previous reports for fiber‐enriched cereals [4]. Overall acceptability was significantly higher for 50% and 75% PSPF formulations, with all samples scoring ≥ 6 on the 9‐point hedonic scale, indicating acceptable sensory quality [6, 48].

## 4. Conclusion

The study demonstrated that the BCF/PSPF ratio significantly influences the structural, functional, antioxidant, and sensory properties of ready‐to‐eat flakes. Higher PSPF levels produced more compact matrices with greater density and fracture force, whereas BCF‐rich formulations favored expanded structures and faster milk absorption. Processing increased water and milk absorption and solubility due to starch gelatinization and microstructural changes. PSPF enrichment increased anthocyanin and flavonoid contents, maintaining high antioxidant capacity despite moderate thermal losses. Sensory evaluation showed that formulations containing 50% and 75% PSPF achieved the highest acceptance. These results indicate that intermediate PSPF levels provide an optimal balance between technological performance, antioxidant potential, and sensory quality for functional breakfast cereals. Accordingly, intermediate BCF/PSPF blends appear to be the most promising formulations for the development of functional ready‐to‐eat breakfast flakes, as they combine acceptable physicochemical properties with enhanced antioxidant potential and favorable sensory response.

## Funding

No funding was received for this manuscript.

## Conflicts of Interest

The authors declare no conflicts of interest.

## Data Availability

The data that support the findings of this study are available from the corresponding author upon reasonable request.

## References

[1] Camire M. E. , Camire A. , and Krumhar K. , Chemical and Nutritional Changes in Foods During Extrusion, Critical Reviews in Food Science and Nutrition. (1990) 29, no. 1, 35–57, 10.1080/10408399009527513. 2184829

[2] Rodríguez-Miranda J. , Carlos-Isidro A. , Martínez-Sánchez C. E. , Herman-Lara E. , Torruco-Uco J. G. , Santiago-Adame R. , and Hernández-Santos B. , Phytochemical Screening, Polyphenols Content and Antioxidant Activity of By-Products of Two Corn Variety, Emirates Journal of Food and Agriculture. (2022) 35, 806–814, 10.9755/ejfa.2022.v34.i10.2930.

[3] Hernández-Santos B. , Lerdo-Reyes A. A. , Téllez-Morales J. A. , and Rodríguez-Miranda J. , Chemical Composition, Techno-Functional Properties, and Bioactive Components of Blends of Blue Corn/Purple Sweet Potato for Its Possible Application in the Food Industry, Journal of Food Measurement and Characterization. (2023) 17, no. 2, 1909–1920, 10.1007/s11694-022-01767-7.

[4] Ding Q. B. , Ainsworth P. , Plunkett A. , Tucker G. , and Marson H. , The Effect of Extrusion Conditions on the Functional and Physical Properties of Wheat-Based Expanded Snacks, Journal of Food Engineering. (2006) 73, no. 2, 142–148, 10.1016/j.jfoodeng.2005.01.013.

[5] Escobar-Puentes A. A. , González-Aguilar G. A. , Olivas-Aguirre F. J. , and Wall-Medrano A. , Sweet Potato (*Ipomoea batatas* L.) Phenotypes: From Agroindustry to Health Effects, Foods. (2022) 11, no. 7, 10.3390/foods11071058, 35407143. PMC8997864 35407143

[6] Jati I. R. A. P. , Darmoatmodjo L. M. Y. D. , Suseno T. I. P. , Ristiarini S. , and Wibowo C. , Effect of Processing on Bioactive Compounds, Antioxidant Activity, Physicochemical, and Sensory Properties of Orange Sweet Potato, Red Rice, and Their Application for Flake Products, Plants. (2022) 11, no. 3, 10.3390/plants11030440, 35161419. PMC8838036 35161419

[7] Nurdjanah S. , Nurdin S. U. , Astuti S. , and Manik V. E. , Chemical Components, Antioxidant Activity, and Glycemic Response Values of Purple Sweet Potato Products, International Journal of Food Science. (2022) 2022, 7708172, 10.1155/2022/7708172, 35465219. 35465219 PMC9033360

[8] Wang X. , Cheng L. , Gu Z. , Hong Y. , Li Z. , Li C. , and Ban X. , Effects of Different Gelatinization Degrees of Potato Flour on Gluten Network Integrity and Dough Stickiness, LWT – Food Science and Technology. (2022) 153, 112577, 10.1016/j.lwt.2021.112577.

[9] Yang J. , Chen J. F. , Zhao Y. Y. , and Mao L. C. , Effects Of Drying Processes on the Antioxidant Properties in Sweet Potatoes, Agricultural Sciences in China. (2010) 9, no. 10, 1522–1529, 10.1016/S1671-2927(09)60246-7.

[10] Horwitz W. and Latimer G. W. , AOAC, Association of Official Analytical Chemists, Official Methods of Analysis, 2015, 19th edition, AOAC International.

[11] Hernández-Santos B. , Quijano-Jerónimo O. , and Rodríguez-Miranda J. , Physical, Chemical, Tecno-Functional, And Thermal Properties of *Leucaena leucocephala* Seed, Food Science and Technology. (2022) 42, e74921, 10.1590/fst.74921.

[12] Borges-Contreras B. , Martínez-Sánchez C. E. , Herman-Lara E. , Rodríguez-Miranda J. , Hernández-Santos B. , Juárez-Barrientos J. M. , Guerra-Almonacid C. M. , Betancur-Ancona D. A. , and Torruco-Uco J. G. , Angiotensin-Converting Enzyme Inhibition In Vitro by Protein Hydrolysates and Peptide Fractions From Mojarra of Nile Tilapia (*Oreochromis niloticus*) Skeleton, Journal of Medicinal Food. (2019) 22, no. 3, 286–293, 10.1089/jmf.2018.0163, 30835154. 30835154

[13] Navarro-Cortez R. O. , Hernández-Santos B. , Gómez-Aldapa C. A. , Castro-Rosas J. , Herman-Lara E. , Martínez-Sánchez C. E. , Juárez-Barrientos J. M. , Antonio-Cisneros C. M. , and Rodríguez-Miranda J. , Development of Extruded Ready-to-Eat Snacks Using PUMPKINSEED (*Cucurbita pepo*) And Nixtamalized Maize (*Zea mays*) Flour Blends, Revista Mexicana de Ingeniería Química. (2016) 15, no. 2, 409–422, 10.24275/rmiq/Alim1135.

[14] Özer E. A. , İbanoğlu Ş. , Ainsworth P. , and Yağmur C. , Expansion Characteristics of a Nutritious Extruded Snack Food Using Response Surface Methodology, European Food Research and Technology. (2004) 218, no. 5, 474–479, 10.1007/s00217-004-0884-7.

[15] Rodríguez-Miranda J. , Gomez-Aldapa C. A. , Castro-Rosas J. , Ramírez-Wong B. , Vivar-Vera M. A. , Morales-Rosas I. , Medrano-Roldan H. , and Delgado E. , Effect Of Extrusion Temperature, Moisture Content and Screw Speed on the Functional Properties of Aquaculture Balanced Feed, Emirates Journal of Food and Agriculture. (2014) 26, no. 8, 10.9755/ejfa.v26i8.17133.

[16] Gan Q. I. U. , Jiang Y. L. , and Yun D. E. N. G. , Drying Characteristics, Functional Properties and In Vitro Digestion of Purple Potato Slices Dried by Different Methods, Journal of Integrative Agriculture. (2019) 18, no. 9, 2162–2172, 10.1016/S2095-3119(19)62654-7.

[17] Ma X. , Jin Z. , and Jin T. , Effects of Extrusion Conditions on Chemical Properties of Extruded White Ginseng Root Hair, Journal of the Science of Food and Agriculture. (2019) 99, no. 6, 3186–3191, 10.1002/jsfa.9535, 30548606. 30548606

[18] Rodrígue-Miranda J. , Rivadeneyra-Rodríguez J. M. , Ramírez-Rivera J. , Juárez-Barrientos J. M. , Herrera-Torres E. , Navarro-Cortez R. O. , and Hernández-Santos B. , Financial, Functional and Content Characteristics of Harina De Malanga (*Colocasia esculenta*), Cultivated in the Tuxtepec Region, Oaxaca, Mexico, Science and Sea. (2011) 15, 37–47.

[19] Re R. , Pellegrini N. , Proteggente A. , Pannala A. , Yang M. , and Rice-Evans C. , Antioxidant Activity Applying an Improved ABTS Radical Cation Decolorization Assay, Free Radical Biology and Medicine. (1999) 26, no. 9-10, 1231–1237, 10.1016/S0891-5849(98)00315-3, 10381194. 10381194

[20] Ying D. , Cheng L. J. , Chibracq G. , Sanguansri L. , Oiseth S. K. , and Augustin M. A. , The Format of *Β*-Carotene Delivery Affects Its Stability During Extrusion, LWT-Food Science and Technology. (2015) 60, no. 1, 1–7, 10.1016/j.lwt.2014.09.034.

[21] Pensamiento-Niño C. A. , Gómez-Aldapa C. A. , Hernández-Santos B. , Juárez-Barrientos J. M. , Herman-Lara E. , Martínez-Sánchez C. E. , Torruco-Uco J. G. , and Rodríguez-Miranda J. , Optimization and Characterization of an Extruded Snack Based on Taro Flour (*Colocasia esculenta* L.) Enriched With Mango Pulp (*Mangifera indica* L.), Journal of Food Science and Technology. (2018) 55, no. 10, 4244–4255, 10.1007/s13197-018-3363-z, 30228423. 30228423 PMC6133830

[22] Cuevas-Montilla E. , Hillebrand S. , Antezana A. , and Winterhalter P. , Soluble and Bound Phenolic Compounds in Different Bolivian Purple Corn (*Zea mays L.*) Cultivars, Journal of Agricultural and Food Chemistry. (2011) 59, no. 13, 7068–7074, 10.1021/jf201061x, 21639140. 21639140

[23] Al-Okbi S. Y. , Hussein A. M. , Hamed I. M. , Mohamed D. A. , and Helal A. M. , Chemical, Rheological, Sensorial and Functional Properties of Gelatinized Corn-Rice Bran Flour Composite Corn Flakes and Tortilla Chips, Journal of Food Processing and Preservation. (2014) 38, no. 1, 83–89, 10.1111/j.1745-4549.2012.00747.x.

[24] Fasuan T. O. , Asadu K. C. , Anyiam C. C. , Ojokoh L. O. , Olagunju T. M. , Chima J. U. , and Okpara K. O. , Bioactive And Nutritional Characterization of Modeled and Optimized Consumer-Ready Flakes From Pseudocereal (*Amaranthus viridis*), High-Protein Soymeal and Modified Corn Starch, Food Production, Processing and Nutrition. (2021) 3, no. 1, 1–13, 10.1186/s43014-021-00057-x.

[25] Fitriani V. , Permana L. , and Setiaboma W. , Chemical and Physical Charaterization of Cereal Flakes Formulated With Broken Rice and Banana Flour, In IOP Conference Series: Earth and Environmental Science. (2019) 258, no. 1, 012003, 10.1088/1755-1315/258/1/012003.

[26] Hu X. Z. , Zheng J. M. , Li X. P. , Xu C. , and Zhao Q. , Chemical Composition and Sensory Characteristics of Oat Flakes: A Comparative Study of Naked Oat Flakes From China and Hulled Oat Flakes From Western Countries, Journal of Cereal Science. (2014) 60, no. 2, 297–301, 10.1016/j.jcs.2014.05.015.

[27] Kim J. T. , Chung I. M. , Kim M. J. , Lee J. S. , Son B. Y. , Bae H. H. , Go Y. S. , Kim S. L. , Baek S. B. , Kim S. H. , and Yi G. , Comparison of Antioxidant Activity Assays in Fresh Purple Waxy Corn (*Zea mays* L.) During Grain Filling, Applied Biological Chemistry. (2022) 65, no. 1, 1–7, 10.1186/s13765-021-00671-w.

[28] Jia R. , Tang C. , Chen J. , Zhang X. , and Wang Z. , Total Phenolics and Anthocyanins Contents and Antioxidant Activity in Four Different Aerial Parts of Leafy Sweet Potato (*Ipomoea batatas* L.), Molecules. (2022) 27, no. 10, 10.3390/molecules27103117, 35630594. PMC9146295 35630594

[29] Castañeda-Ovando A. , Pacheco-Hernández M. L. , Páez-Hernández M. E. , Rodríguez J. A. , and Galán-Vidal C. A. , Chemical Studies of Anthocyanins: A Review, Food Chemistry. (2009) 113, no. 4, 859–871, 10.1016/j.foodchem.2008.09.001.

[30] Khoo H. E. , Azlan A. , Tang S. T. , and Lim S. M. , Anthocyanidins and Anthocyanins: Colored Pigments as Food, Pharmaceutical Ingredients, and the Potential Health Benefits, Food & Nutrition Research. (2017) 61, no. 1, 1361779, 10.1080/16546628.2017.1361779, 28970777. 28970777 PMC5613902

[31] Bach D. , Bedin A. C. , Lacerda L. G. , Nogueira A. , and Demiate I. M. , Sweet Potato (*Ipomoea batatas L.*): A Versatile Raw Material for the Food Industry, Brazilian Archives of Biology and Technology. (2021) 64, e21200568, 10.1590/1678-4324-2021200568.

[32] Singh N. , Singh J. , Kaur L. , Sodhi N. S. , and Gill B. S. , Morphological, Thermal and Rheological Properties of Starches From Different Botanical Sources, Food Chemistry. (2003) 81, no. 2, 219–231, 10.1016/S0308-8146(02)00416-8.

[33] Jane J. L. , Structural Features of Starch Granules II, Starch, 2009, Academic Press, 193–236, 10.1016/B978-0-12-746275-2.00006-9.

[34] Hoover R. , Composition, Molecular Structure, and Physicochemical Properties of Tuber and Root Starches: A Review, Carbohydrate Polymers. (2001) 45, no. 3, 253–267, 10.1016/S0144-8617(00)00260-5.

[35] Gujska E. and Khan K. , High Temperature Extrusion Effects on Protein Solubility and Distribution in Navy and Pinto Beans, Journal of Food Science. (1991) 56, 1013–1016, 10.1111/j.1365-2621.1991.tb14629.x.

[36] Zhu F. and Wang S. , Physicochemical Properties, Molecular Structure, and Uses of Sweetpotato Starch, Trends in Food Science & Technology. (2014) 36, no. 2, 68–78, 10.1016/j.tifs.2014.01.008.

[37] Fuentes-Zaragoza E. , Sánchez-Zapata E. , Sendra E. , Sayas E. , Navarro C. , Fernández-López J. , and Pérez-Alvarez J. A. , Resistant Starch as Prebiotic: A Review, Starch/Stärke. (2011) 63, no. 7, 406–415, 10.1002/star.201000099.

[38] Escalante-Aburto A. , Ramírez-Wong B. , Torres-Chávez P. I. , Figueroa-Cárdenas J. D. , López-Cervantes J. , Barrón-Hoyos J. M. , and Morales-Rosas I. , Effect of Extrusion Processing Parameters on Anthocyanin Content and Physicochemical Properties of Nixtamalized Blue Corn Expanded Extrudates, CyTA-Journal of Food. (2013) 11, no. sup1, 29–37, 10.1080/19476337.2013.764929.

[39] Camacho-Hernández I. L. , Zazueta-Morales J. J. , Gallegos-Infante J. A. , Aguilar-Palazuelos E. , Rocha-Guzmán N. E. , Navarro-Cortez R. O. , Jacobo-Valenzuela N. , and Gómez-Aldapa C. A. , Effect of Extrusion Conditions on Physicochemical Characteristics and Anthocyanin Content of Blue Corn Third-Generation Snacks, CyTA-Journal of Food. (2014) 12, no. 4, 320–330, 10.1080/19476337.2013.861517.

[40] Özer E. A. , Herken E. N. , Güzel S. , Ainsworth P. , and İbanoğlu Ş. , Effect of Extrusion Process on the Antioxidant Activity and Total Phenolics in a Nutritious Snack Food, International Journal of Food Science and Technology. (2006) 41, no. 3, 289–293, 10.1111/j.1365-2621.2005.01062.x.

[41] Zhao S. , Zhong L. , Li X. , Qin L. , Zhou Y. , Lei X. , Zheng X. , Jin K. , Pu Z. , Hou X. , Lang T. , Zhang C. , and Feng J. , Comparative Analysis of Nutrients, Phytochemicals, and Minerals in Colored Sweet Potato (*Ipomoea batatas* L.) Roots, Foods. (2024) 13, no. 22, 10.3390/foods13223636, 39594052. PMC11593716 39594052

[42] Dewanto V. , Wu X. , Adom K. K. , and Liu R. H. , Thermal Processing Enhances the Nutritional Value of Tomatoes by Increasing Total Antioxidant Activity, Journal of Agricultural and Food Chemistry. (2002) 50, no. 10, 3010–3014, 10.1021/jf0115589, 11982434. 11982434

[43] Arribas C. , Cabellos B. , Cuadrado C. , Guillamon E. , and Pedrosa M. M. , The Effect of Extrusion on the Bioactive Compounds and Antioxidant Capacity of Novel Gluten-Free Expanded Products Based on Carob Fruit, Pea and Rice Blends, Innovative Food Science & Emerging Technologies. (2019) 52, 100–107, 10.1016/j.ifset.2018.12.003.

[44] Wani S. A. , Ganie N. A. , and Kumar P. , Quality Characteristics, Fatty Acid Profile and Glycemic Index of Extrusion Processed Snacks Enriched With the Multicomponent Mixture of Cereals and Legumes, Legume Science. (2021) 3, no. 2, 10.1002/leg3.76.

[45] Neder-Suárez D. , Quintero-Ramos A. , Meléndez-Pizarro C. O. , de Jesús Zazueta-Morales J. , Paraguay-Delgado F. , and Ruiz-Gutiérrez M. G. , Evaluation of the Physicochemical Properties of Third-Generation Snacks Made From Blue Corn, Black Beans, and Sweet Chard Produced by Extrusion, LWT - Food Science and Technology. (2021) 146, 111414, 10.1016/j.lwt.2021.111414.

[46] Teow C. C. , Truong V. D. , McFeeters R. F. , Thompson R. L. , Pecota K. V. , and Yencho G. C. , Antioxidant Activities, Phenolic and *Β*-Carotene Contents of Sweet Potato Genotypes With Varying Flesh Colours, Food Chemistry. (2007) 103, no. 3, 829–838, 10.1016/j.foodchem.2006.09.033.

[47] Makori S. I. , Mu T. H. , and Sun H. N. , Total Polyphenol Content, Antioxidant Activity, and Individual Phenolic Composition of Different Edible Parts of 4 Sweet Potato Cultivars, Natural Product Communications. (2020) 15, no. 7, 1934578X20936931, 10.1177/1934578X20936931.

[48] Delgado-Licon E. , Ayala A. L. M. , Rocha-Guzman N. E. , Gallegos-Infante J. A. , Atienzo-Lazos M. , Drzewiecki J. , Martínez-Sanchez C. E. , and Gorinstein S. , Influence of Extrusion on the Bioactive Compounds and the Antioxidant Capacity of the Bean/Corn Mixtures, International Journal of Food Sciences and Nutrition. (2009) 60, no. 6, 522–532, 10.1080/09637480801987666, 18608556. 18608556

